# Effectiveness of psychological first aid in infectious disease pandemics: An overview of systematic reviews

**DOI:** 10.1002/pcn5.107

**Published:** 2023-06-08

**Authors:** Masahide Koda, Toru Horinouchi, Nozomu Oya, Morio Aki, Akihisa Iriki, Kazufumi Yoshida, Yusuke Ogawa, Hironori Kuga, Tomohiro Nakao

**Affiliations:** ^1^ Co‐Learning Community Healthcare Re‐Innovation Office Graduate School of Medicine Okayama Japan; ^2^ Department of Psychiatry Hokkaido University Graduate School of Medicine Hokkaido Japan; ^3^ Department of Psychiatry, Graduate School of Medical Science Kyoto Prefectural University of Medicine Kyoto Japan; ^4^ Department of Psychiatry, Graduate School of Medicine Kyoto University Kyoto Japan; ^5^ Osaka Psychiatric Medical Center Osaka Japan; ^6^ Department of Health Promotion and Human Behavior, Graduate School of Medicine/School of Public Health Kyoto University Kyoto Japan; ^7^ Department of Healthcare Epidemiology, School of Public Health in the Graduate School of Medicine Kyoto University Kyoto Japan; ^8^ National Center for Cognitive Behavior Therapy and Research National Center of Neurology and Psychiatry Tokyo Japan; ^9^ Department of Neuropsychiatry, Graduate School of Medical Sciences Kyushu University Fukuoka Japan

**Keywords:** mental health, pandemic, psychological first aid, psychosocial support

## Abstract

There is insufficient research on the usefulness of psychological interventions, such as psychological first aid (PFA), during outbreaks. We searched for and critically appraised systematic reviews that examined the effectiveness of PFA during infectious disease outbreaks, such as the novel coronavirus disease (COVID‐19). Systematic reviews that examined the efficacy of PFA in the severe acute respiratory syndrome, Middle East respiratory syndrome coronavirus, Ebola virus disease, and COVID‐19 outbreaks were searched through PubMed on February 19, 2021. The three included systematic reviews were critically appraised and assessed using AMSTAR‐2. One review's overall confidence in its findings was evaluated as “high,” which suggested that PFA training had a favorable effect on healthcare personnel. Furthermore, the review also demonstrated that PFA was commonly used during outbreaks and could be delivered through multiple methods, such as a phone or video call. Although it was anticipated that PFA would improve subjective well‐being, reports showed no evidence of reduced depression or insomnia. Future studies should examine additional numbers of PFA recipients and conduct quasi‐experimental studies to better understand the effectiveness of PFA. Evidence on its effectiveness in infectious disease outbreaks is still lacking, along with research and evaluation methods. Quasi‐experimental studies, such as comparisons with other psychological interventions, are required to better understand the effectiveness of PFA.

## Graphical abstract

We aimed to examine the effectiveness of psychological first aid (PFA) in epidemics and overviewed the existing systematic reviews of PFA in outbreaks, such as the novel coronavirus disease (COVID‐19). As a result, we found three relevant articles, and one had a confidence “high” rating on the AMSTAR‐2, which suggested that PFA training positively affected healthcare providers.

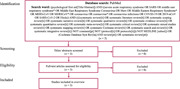

## INTRODUCTION

Infectious diseases outbreaks can significantly impact the mental health of individuals who are infected. Psychological distress due to exposure to infectious diseases can cause various mental health problems, including anxiety, depression, and post‐traumatic stress disorder, in both the general population and healthcare workers.^1^, ^2^, ^3^ Additionally, the tension, fear, and anxiety caused by an outbreak can disrupt civil society and have adverse economic, political, psychological, and physical effects.^4^, ^5^ Given the potentially serious effects of psychological distress during an infectious disease outbreak, interventions that alleviate psychological discomfort and encourage adaptive coping mechanisms are necessary. We chose psychological first aid (PFA) as the focus of this review owing to its unique potential for widespread implementation, particularly during a global crisis, such as the coronavirus disease (COVID‐19) pandemic. Unlike some other psychological interventions, PFA can be delivered by various individuals, including nonprofessionals, after receiving appropriate training. This flexibility makes PFA especially valuable in situations where mental health resources may be scarce or inaccessible. Moreover, it is designed to address immediate psychological needs and foster resilience, crucial aspects of mental health support during infectious disease outbreaks.^6^

PFA, an intervention to alleviate psychological discomfort,^7^, ^8^, ^9^, ^10^ offers compassion and help to people in need who have survived a natural disaster or catastrophic economic crisis. It involves a range of activities, including listening, offering consolation, and facilitating interpersonal connections. Furthermore, it is open to both the general population and mental health professionals. Research on PFA interventions in general disasters, excluding infectious diseases, has shown mixed results, with some studies reporting positive outcomes, such as reduced psychological distress.^6^ In contrast, other studies report limited or inconclusive evidence of its effectiveness.^11^ Given these inconsistent findings, examining the effectiveness of PFA during infectious disease outbreaks, such as the COVID‐19 pandemic, is important for crisis‐response strategies and optimizing psychological support for the affected populations.

However, the extent to which PFA was effective during an infectious disease outbreak was unknown. The worldwide COVID‐19 pandemic increased stress and anxiety levels, even though its long‐term effects on stress levels were unclear.^10^, ^11^, ^12^ Social isolation, due to the COVID‐19 pandemic, has had a significant psychological impact on people globally.^4,5^ Therefore, this study aimed to determine the value of PFA in an infectious disease pandemic and whether it contributed against mental illness caused by the COVID‐19 pandemic.

## METHODS

We conducted a literature search using the overview method and searched the PubMed database on February 19, 2021 for articles on the effectiveness of PFA, regardless of language or publication year.^13^ An overview of systematic reviews was selected as it considered the broad range of available evidence, could assess the risk of bias, and provide a comprehensive perspective.^14^ While systematic reviews answered specific research questions, overviews offered a further extensive examination of the existing literature, which we deemed was more suitable for the current research on PFA during infectious disease outbreaks, which included the COVID‐19 pandemic. We aimed to examine multiple systematic reviews, synthesize the diverse evidence available, identify gaps in knowledge, and provide a further informed foundation for future research on PFA in this context. Studies were included if they mentioned “psychological first aid” in either the title or abstract and were related to major infectious disease outbreaks since 2000. There are several existing PFA models, such as the World Health Organization's PFA (WHO‐PFA), the National Child Traumatic Stress Network PFA (NCTSN‐PFA), and the Johns Hopkins Guide to PFA (RAPID‐PFA) model.^15^ These models aimed to provide immediate support to individuals who were distressed; however, they differed in their specific approaches and guidelines. In this overview, we conducted a search, regardless of the type of PFA employed, to gain a broader understanding of its effectiveness during infectious disease outbreaks. A pandemic was classified as an outbreak that affected people worldwide, crossed international borders, and involved many people.^16^ The severe acute respiratory syndrome (SARS), Middle East respiratory syndrome (MERS), Ebola virus disease (EVD), and COVID‐19 outbreaks were included. The complete search list is shown in Table 1.

**Table 1:** Search syntax.

(psychological first aid[Title/Abstract]) AND ((severe acute respiratory syndrome OR SARS OR middle east respiratory syndrome* OR Middle East Respiratory Syndrome Coronavirus OR Mers OR Middle Eastern Respiratory Syndrome* OR MERS‐CoV OR MERSCoV* OR coronavirus OR coronavirus* OR coronavirus infections OR COVID‐19 OR 2019‐nCoV OR SARS‐CoV‐2 OR Ebola)) AND ((((systematic review[ti] OR systematic literature review[ti] OR systematic scoping review[ti] OR systematic narrative review[ti] OR systematic qualitative review[ti] OR systematic evidence review[ti] OR systematic quantitative review[ti] OR systematic meta‐review[ti] OR systematic critical review[ti] OR systematic mixed studies review[ti] OR systematic mapping review[ti] OR systematic Cochrane review[ti] OR systematic search and review[ti] OR systematic integrative review[ti]) NOT comment[pt] NOT (protocol[ti] OR protocols[ti])) NOT MEDLINE [subset]) OR (Cochrane Database Syst Rev[ta] AND review[pt]) OR systematic review[pt]))))

Abbreviations: 2019‐nCoV, 2019 novel coronavirus; COVID‐19, coronavirus disease 2019; MERS‐CoV, Middle East respiratory syndrome coronavirus; SARS, severe acute respiratory syndrome; SARS‐CoV‐2, severe acute respiratory syndrome coronavirus‐2.

To incorporate reproducible methods, we included systematic reviews of randomized controlled trials (RCTs) or prospective or retrospective cohort studies. We also accepted qualitative reports, with or without meta‐analysis, and did not have language restrictions. This study did not consider the participants' sexes, nationalities, or races. Before the review began, psychological interventions were deemed to have been performed in one of the following infectious disease environments: SARS, MERS, EVD, or COVID‐19. Therefore, we also included other psychological interventions. M.A. confirmed the methods, M.K. and T.H. performed the screening, and A.I. extracted data. M.K. and K.Y. independently evaluated the studies via AMSTAR‐2.^17^ The Preferred Reporting Items of Systematic Reviews and Meta‐Analysis (PRISMA) guidelines were followed.^18^ An ethical review was not required since this was a secondary data study.

## RESULTS

**Figure 1:**
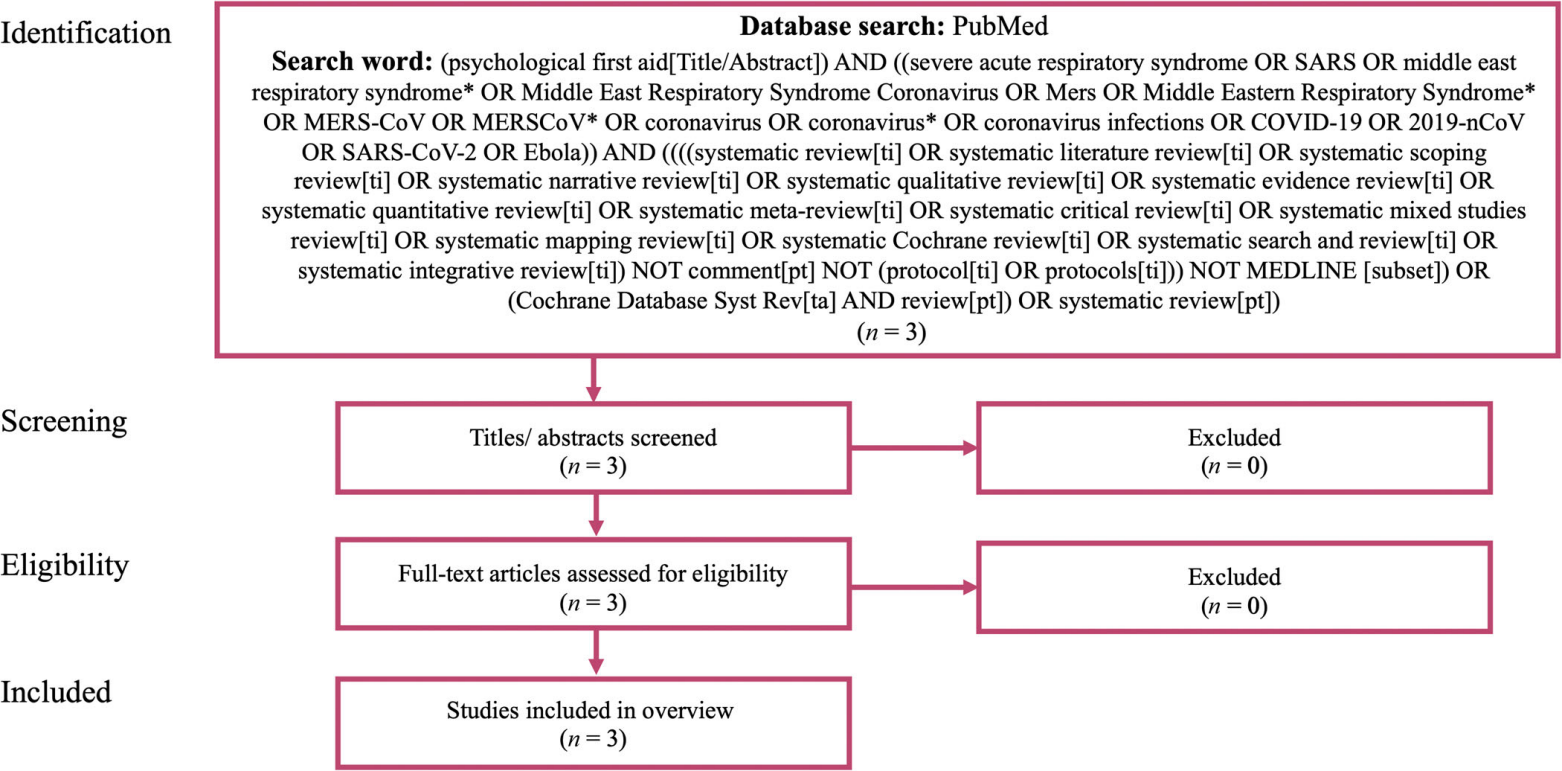
Flow chart. The PubMed database was systematically searched on February 19, 2021, using the terms “psychological first aid” in the title or abstract, restricted to articles published since 2000 related to major infectious disease outbreaks. In total, three systematic reviews were examined for eligibility and judged to be consistent with the study's objectives.

The flowchart of this study is shown in Figure 1. In total, three systematic reviews were identified during the screening phase. After an evaluation for their eligibility, all were judged consistent with the study's objectives.

A critical appraisal of the review articles using the AMSTAR‐2 is provided in Table 2. Review article (1) Pollock et al.^19^ was rating “high,” which implied that the findings were accurately and comprehensively summarized. However, the other two review articles, (2) Yue et al.,^20^ and (3) Cénat et al.,^21^ were rated “critically low” as one or more fatal flaws existed. Hence, they were deemed unreliable for providing an accurate and comprehensive summary.

**Table 2:** The AMSTAR‐2 assessment.

SRs	Item																Overall
	1	2^a^	3	4^a^	5	6	7^a^	8	9^a^	10	11^a^	12	13^a^	14	15^a^	16	quality
**Pollock et al**.^17^	**Y**	**Y**	**Y**	**Y**	**Y**	**Y**	**Y**	**Y**	**Y**	**Y**	NMC	NMC	**Y**	**Y**	NMC	**Y**	High
**Yue et al**.^18^	**Y**	N	**Y**	PY	**Y**	**Y**	N	PY	PY	N	NMC	NMC	N	**Y**	NMC	**Y**	Critically low
**Cénat et al**.^19^	**Y**	N	**Y**	N	N	N	N	PY	N	**Y**	NMC	NMC	N	**Y**	NMC	**Y**	Critically low

*Notes*: Item 1, “Did the research questions and inclusion criteria include components of the clinical question (patients, intervention, comparison, and outcome)?”; Item 2, “Did the report contain an explicit statement that the review methods were established prior to the review being conducted and justify any significant protocol deviations?”; Item 3, “Did the authors explain their selection of the studies for inclusion?”; Item 4, “Did they use a comprehensive literature search strategy?”; Item 5, “Did they perform study selection in duplicate?”; Item 6, “Did they perform data extraction in duplicate?”; Item 7, “Did they provide a list of excluded studies and justify them?”; Item 8, “Were the included studies described in adequate detail?”; Item 9, “Was a satisfactory technique used to assess the risk of bias (RoB) in the individual included studies?”; Item 10, “Did they report the sources of funding for the studies included?”; Item 11, “If a meta‐analysis was performed, did they use appropriate methods for statistical combination of the results?”; Item 12, “If a meta‐analysis was performed, did they assess the potential impact of the RoB in individual studies on the results of the meta‐analysis or other evidence synthesis?”; Item 13, “Did they account for the RoB in individual studies during the interpretation/discussion of the results?”; Item 14, “Did they provide a satisfactory explanation for and discussion of any heterogeneity in the results?”; Item 15, “If they performed quantitative synthesis, was an adequate investigation of publication bias (small study bias) conducted and its likely impact on the results discussed?”; Item 16 “Did they report any potential sources of conflict of interest, including any funding they received for conducting the review?”

Abbreviations: N, no; NMC, no meta‐analysis conducted; PY, partial yes; Y, yes.

^a^ Critical domains.

In total, 16 studies were included in Pollock et al.,^19^, ^22^, ^23^, ^24^, ^25^, ^26^, ^27^, ^28^, ^29^, ^30^, ^31^, ^32^, ^33^, ^34^, ^35^, ^36^, ^37^ 32 in Yue et al.,^20^, ^35^, ^36^, ^37^, ^38^, ^39^, ^40^, ^41^, ^42^, ^43^, ^44^, ^45^, ^46^, ^47^, ^48^, ^49^, ^50^, ^51^, ^52^, ^53^, ^54^, ^55^, ^56^, ^57^, ^58^, ^59^, ^60^, ^61^, ^62^, ^63^, ^64^, ^65^, ^66^, ^67^, ^68^ and 11 in Cénat et al.^21^, ^65^, ^66^, ^67^, ^68^, ^69^, ^70^, ^71^, ^72^, ^73^, ^74^, ^75^ All the reviews included PFA as an intervention; however, various other interventions, such as cognitive behavioral therapy (CBT) and counseling, were also practiced. (1) Pollock et al.^19^ and (2) Yue et al.^20^ included the COVID‐19 pandemic, while (3) Cénat et al.^21^, ^65^, ^66^, ^67^, ^68^, ^69^, ^70^, ^71^, ^72^, ^73^, ^74^, ^75^ dealt only with the Ebola outbreak. Populations targeted by the interventions varied between (2) Yue et al.^20^ and (3) Cénat et al.,^21^ which included various people, while Pollock et al.^19^ only included healthcare workers who worked at the front lines (Table 3).

**Table 3:** Overview of the key characteristics of the included reviews.

	Included databases	Search date	Included study design	A limited selection of studies (if any)	Infectious disease	Target population	Mental healthcare/psychological intervention	Outcomes
**Pollock et al**.^17^	Cochrane Database of Systematic Reviews, Global Index Medicus databases, CENTRAL, MEDLINE, Embase, Web of Science, PsycINFO, CINAHL, WHO Institutional Repository for Information Sharing, Trials Registrations, and Google Scholar	May 28, 2020	One mixed‐method design described the implementation and evaluation of an intervention and included a qualitative interview component and a cohort study, while two quantitative study designs included some descriptive data. Six qualitative study designs, one mixed‐method design that reported results from quantitative questionnaires and qualitative interviews, one that described the implementation and evaluation of an intervention, and five that did both.	All articles from 2002 onwards, with no language restrictions.	Four COVID‐19 Nine EVD One MERS Two SARS	Health and social care professionals who worked at the front line during infectious disease outbreaks (epidemics and pandemics).	Interventions at the workplace, including training, structure, and communication (six studies); approaches for psychological support, including counseling and psychological services (eight studies); and multimodal interventions (two studies).	General mental health and resilience, psychological signs of anxiety, depression, or stress, burnout, other mental health disorders, workplace staffing, and adverse events resulting from the interventions were examined to determine the impact of work‐based interventions. The results provided very low‐certainty evidence regarding the impact of training frontline healthcare workers to provide psychological first aid on a measure of burnout.
**Yue et al**.^18^	PubMed, Web of Science, Embase, PsycINFO, WHO Global Database on COVID‐19, and MedRxiv	May 5, 2020	One RCT, one quasi‐experimental intervention, four pre–post interventions, one intervention development, one quantitative interview, eight reports, 14 commentaries, and two reviews.	Excluded non‐English publications	23 COVID‐19 Seven EVD One MERS One SARS	Healthcare workers, psychiatric patients, individuals who were quarantined, family members, older adults, and children.	Group‐based cognitive behavioral therapy, PFA, community‐based psychological art program, and other culturally adapted interventions.	To improve mental health response capacity for ongoing and upcoming infectious disease outbreaks, culturally appropriate, financially viable, and easily accessible strategies integrated into the local and national public health emergency response and established medical systems were likely to be effective. Although tele‐mental healthcare services were important essential elements of care for managing infectious disease outbreaks and providing routine assistance, the value and constraints of distant healthcare delivery should also be acknowledged.
**Cénat et al**.^19^	EMBASE, PsycINFO, PubMed, MEDLINE, Cochrane Library, and PILOTS	Not stated	Any research plan, analysis, or summary of successfully implemented and assessed psychosocial treatments (no statement).	Those launched between 2013 and 2019.	11 EVD	Those who have experienced EVD (survivors, health workers, volunteers, other frontline workers, children, adults, etc.). Two programs focused on impacted children and families, while four programs offered MHPSS to employees and volunteers. Communities and ETCs established other unique programs.	Five programs incorporated the concepts of PFA.	Findings indicated improvements in both children's and adults' mental health. (Culturally tailored MHPSS programs may benefit both adults and children afflicted by EVD and have a positive influence on the relationship between the emotional consequences of EVD and use of preventative measures.)

Abbreviations: COVID‐19, coronavirus disease 2019; ETCs, Ebola treatment centers; EVD, Ebola virus disease; MERS, Middle East respiratory syndrome; MHPSS, mental health and psychosocial support; PFA, psychological first aid; RCTs, randomized controlled trials; SARS, severe acute respiratory syndrome.

## DISCUSSION

This study overviewed three systematic reviews to determine the effectiveness of PFA during infectious disease outbreaks. Although three systematic reviews were identified, only (1) Pollock et al.^19^ had an AMSTAR‐2 overall confidence rating of “high.” Meanwhile (2) Yue et al.,^20^ and (3) Cénat et al.^21^ were rated “critically low.” The AMSTAR‐2 was used to appraise the methodological quality of the identified systematic reviews, which included the Cochrane review by Pollock et al. This evaluation tool was widely recognized for assessing the quality of systematic reviews.^14^ Although the Cochrane review had the highest quality, the inclusion of other systematic reviews, along with their AMSTAR‐2 assessments, provided a further comprehensive understanding of the available evidence on PFA during infectious disease outbreaks. Furthermore, it enabled us to identify the potential biases and limitations in the additional reviews and allowed for a further cautious interpretation of their findings. As a result of possible bias, (2) Yue et al.^20^ and (3) Cénat et al.^21^ should be interpreted with caution. Both failed to develop and report on the review process before the study was executed, did not compile a list of the excluded studies, and failed to apply the bias risk effectively in the interpretation and discussion. However, it was important to consider their findings as they may provide valuable insights on the investigated topic.

In the context of general disasters not related to infectious diseases, the effectiveness of PFA remains debatable. Some studies demonstrated positive outcomes, such as reduced psychological distress and improved coping mechanisms.^6^ However, others yielded limited or inconclusive evidence regarding PFA's effectiveness in these situations.^11^ This inconsistency underscored the need for further investigation and for methodologically rigorous studies to better understand the potential benefits and limitations of PFA interventions across various disaster contexts, which included infectious disease outbreaks, such as the COVID‐19 pandemic.

We included other interventions, such as CBT, to provide a further comprehensive view of the existing literature on psychological interventions during outbreaks and highlight potential alternative or complementary approaches explored in the context of infectious disease outbreaks. Although our primary focus was on PFA, our literature overview revealed that many systematic reviews also discussed other interventions, such as CBT, which we have included in our discussion for a comprehensive understanding of the field.

### PFA

The main goal of the (1) Pollock et al. study^19^ was to evaluate the efficacy of treatments intended to improve resilience among frontline healthcare personnel. The study found that training healthcare workers to practice PFA that measured burnout was effective. However, confidence in the effectiveness was low.^30^ Nevertheless, this study showed that healthcare workers could experience positive changes through PFA techniques. These included an improved ability to understand people's reactions and control one's emotions; improved relationships with friends, family, and colleagues; and development of better self‐care strategies. PFA was also commonly used in other settings, such as in Sierra Leone where 14 nurses were trained in PFA for Ebola,^68^ as well as teams of food providers, contact tracers, and transportation operators to households during quarantine.^72^ In Guinea, 46 traditional healers and 154 imams were trained in PFA and psychosocial support by the Red Cross.^71^ Other interventions included a combination of PFA and group‐based CBT.^67^ However, while PFA was beneficial as an early intervention for individuals in distress, its effectiveness was not always particular. While trainers received training to conduct PFA, this training was often shorter and incomplete compared with the standard.^76^ In the systematic reviews included in our study, the specific PFA model employed was not always clearly identified. However, most studies may have focused on the WHO‐PFA model, given its widespread recognition and use.^15^ The RAPID‐PFA model, a more recent development, has not been as extensively documented, and its effectiveness during infectious disease outbreaks should be further investigated.^10^ Future research should explore the potential benefits and limitations of the different PFA models, including the RAPID‐PFA, to gain a better understanding of their applicability and effectiveness in various contexts.

### CBT

Another strategy supported by evidence was CBT. A study on 253 staff members from an Ebola treatment center found that small‐group CBT delivered in eight sessions over 6 weeks improved staff anxiety, depression, and dysfunction.^37^ Another study, which administered PFA to staff who worked at an Ebola treatment center and CBT intervention to a group that exhibited high anxiety and depression, found that stress, anxiety, depression, and anger all improved. Furthermore, the interventions protected the staff from adverse psychological outcomes. However, they also found that the team lacked the motivation to participate in CBT, which underscored the importance of motivation.^66^, ^67^ Given the availability of online and mobile application platforms in addition to in‐person encounters, CBT has the potential to be accessible to a wide audience.^20^ Some tele‐mental health services were offered in China during the COVID‐19 pandemic, and online self‐help psychotherapy, such as CBT for insomnia and depression, was also provided.^63^

### Other interventions

The SMART group debriefing (Strength‐Focused and Meaning‐Oriented Approach for Resilience and Transformation) included 51 patients who were chronically ill from Hong Kong after the SARS outbreak. Participants' depressive symptoms decreased, and their attitudes regarding SARS underwent an adaptive change.^57^ In addition, during the Ebola epidemic in Sierra Leone, a model was developed to help healthcare workers manage psychological risk and resilience.^34^ The model was based on self‐triage, in which healthcare providers anticipated their own stress experiences, developed a plan to increase resilience, and deterred stress reactions by activating the program when exposed to stress. Self‐triage was a promising method to avoid dysfunction, and preliminary studies showed that it was feasible. Other interventions adapted to different cultures, such as Playing to Live in Liberia,^65^ ultra‐brief psychological intervention in Malaysia,^54^ and peer support among university students in Iran using social media platforms,^55^ also alleviated psychological malaise during infectious disease outbreaks.

### PFA in the context of general disasters

In addition to its use in infectious disease outbreaks, PFA has also been employed in various noninfectious contexts, including natural disasters, terrorist attacks, and other traumatic events. Understanding its effectiveness in these situations can provide valuable insights into its overall utility across different types of crises. Some studies demonstrated the positive impact of PFA on coping, resilience, and mental health outcomes in noninfectious settings.^15^ However, evidence remains limited, especially in the context of infectious disease. Furthermore, methodological issues in the existing literature must be addressed to draw further robust conclusions.^11^

### What providers should be aware of when implementing interventions

According to the high‐quality (1) Pollock et al.^19^ review, there were two reported barriers to intervention for frontline healthcare workers. Healthcare workers and their organizations lacked the necessary knowledge to assist their mental health. Furthermore, healthcare workers lacked the tools, staff time, and expertise required to perform the intervention, as mentioned above.

### Social relations

It is essential to be aware of how society views patients with infectious diseases, as this can significantly influence their hospitalization and reintegration into the community. During the Ebola epidemic, previous studies found that stigma, discrimination, and social rejection were significant problems for patients.^77^, ^78^ Although there has been no particular research on this during the COVID‐19 pandemic, patients could have had comparable problems. Therefore, it is essential to be mindful of this when working with patients with infectious diseases and encourage society to reduce the related stigma.

### Limitations

This study has several limitations. First, the review compiled was limited to studies that implemented PFA interventions, such as PFA for outbreaks. Furthermore, limited studies with substantial evidence, such as RCTs, were identified. Moreover, evaluation methods across studies were heterogeneous. Hence, it was challenging to construct a meta‐analysis or other high level of evidence. Second, a significant challenge was the need for further research on PFA during infectious disease outbreaks and in disaster situations. PFA was anticipated to be a practical approach for mental health and psychosocial support during infectious disease outbreaks; however, there was insufficient evidence to substantiate this approach. Additional evidence should be gathered in the future. Nevertheless, the limited time and resources available during disasters made it difficult to conduct rigorously controlled studies. Consequently, it was essential to further accumulate findings from various research designs, such as quasi‐experimental and controlled intervention studies. Additionally, in regions where English was not the native language, the number of publications in English may be limited, which could result in an evidence gap if only English‐language literature was considered.^79^ Therefore, a comprehensive survey that encompassed non‐English‐language literature should be conducted. Third, a key issue in conducting research during a disaster was ethical considerations.^80^, ^81^ Given a disaster's chaotic and often dangerous nature, researchers are required to take extra care and ensure that the participants understand what they have consented to and that they are not being coerced into participating. Researchers should be sensitive to participants' potential mental and emotional distress and take steps to minimize any risks associated related to their participation. Therefore, quasi‐experimental studies on PFA that consider the ethical aspects of the people who are affected are desirable. Quasi‐experimental studies that compare the results of PFA interventions with other types of interventions, examine medium‐ and long‐term effects, or compare the results of PFA administered by different providers (e.g., mental health professionals or nonprofessionals) would be useful in understanding its multifaceted effects.

## CONCLUSION

PFA is becoming more widely used in infectious disease outbreaks. Although the results of high‐quality systematic reviews are limited, many reports have demonstrated its effectiveness. During implementation, it is essential for providers to be aware of the mental well‐being of COVID‐19 patients and behave in a way that reduces the social stigma attached to them. However, evidence on PFA being an effective tool for mental health and psychosocial support in outbreaks is still insufficient due to lacking studies, designs, and evaluation methods. While considering the ethical considerations of the people who are affected, quasi‐experimental analyses, such as comparisons with other psychological interventions, are required to better understand the effectiveness of PFA.

## AUTHOR CONTRIBUTIONS

Masahide Koda, Toru Horinouch, and Nozomu Oya designed the study. Masahide Koda and Toru Horinouchi wrote the first draft of the manuscript. Masahide Koda, Toru Horinouchi, Nozomu Oya, Morio Aki, Akihisa Iriki, Kazufumi Yoshida, and Yusuke Ogawa performed a literature search and analysis. Masahide Koda and Kazufumi Yoshida evaluated the past systematic reviews via the AMSTAR‐2. All the authors made a substantial contribution to the manuscript, and approved of the final draft.

## CONFLICT OF INTEREST STATEMENT

Masahide Koda received a Grant‐in‐Aid for Early‐Career Scientists (JP19K19462) from the Japan Society for the Promotion of Science (JSPS), and a medical research grant from Pfizer Health Research Foundation. Toru Horinouch received a scholarship to study abroad from the Japan Epilepsy Research Foundation. Morio Aki received honoraria for lectures from Sumitomo Pharma Co., Ltd. Nozomu Oya, Akihisa Iriki, Kazufumi Yoshida, Yusuke Ogawa, Hironori Kuga, and Tomohiro Nakao have nothing to disclose. The authors declare no conflicts of interest.

## ETHICS APPROVAL STATEMENT

Since this study was a review, ethical approval was not necessary. This study followed the PRISMA guidelines.

## PATIENT CONSENT STATEMENT

None.

## CLINICAL TRIAL REGISTRATION

None.

## Supporting information

Supporting information.

## Data Availability

None.

## References

[1] Luo Y , Chua CR , Xiong Z , Ho RC , Ho CSH . A systematic review of the impact of viral respiratory epidemics on mental health: an implication on the coronavirus disease 2019 pandemic. Front Psychiatry. 2020;11:565098. 10.3389/fpsyt.2020.565098 33329106 PMC7719673

[2] Preti E , Di Mattei V , Perego G , Ferrari F , Mazzetti M , Taranto P , et al. The psychological impact of epidemic and pandemic outbreaks on healthcare workers: rapid review of the evidence. Curr Psychiatry Rep. 2020;22:43. 10.1007/s11920-020-01166-z 32651717 PMC7350408

[3] Serrano‐Ripoll MJ , Meneses‐Echavez JF , Ricci‐Cabello I , Fraile‐Navarro D , Fiol‐deRoque MA , Pastor‐Moreno G , et al. Impact of viral epidemic outbreaks on mental health of healthcare workers: a rapid systematic review and meta‐analysis. J Affect Disord. 2020;277:347–57. 10.1016/j.jad.2020.08.034 32861835 PMC7443314

[4] Pietrabissa G , Simpson SG . Psychological consequences of social isolation during COVID‐19 outbreak. Front Psychol. 2020;11:2201. 33013572 10.3389/fpsyg.2020.02201 PMC7513674

[5] Torales J , O′Higgins M , Castaldelli‐Maia JM , Ventriglio A . The outbreak of COVID‐19 coronavirus and its impact on global mental health. Int J Soc Psychiatry. 2020;66:317–20. 10.1177/0020764020915212 32233719

[6] Shah K , Bedi S , Onyeaka H , Singh R , Chaudhari G . The role of psychological first aid to support public mental health in the COVID‐19 pandemic. Cureus. 2020;12:8821. 10.7759/cureus.8821 PMC7384717 32742836

[7] Ruzek JI , Brymer MJ , Jacobs AK , Layne CM , Vernberg EM , Watson PJ . Psychological first aid. J Ment Health Couns. 2007;29:17–49. 10.17744/mehc.29.1.5racqxjueafabgwp

[8] World Health Organization . Psychological first aid: guide for field workers. Geneva: World Health Organization; 2011. https://www.who.int/publications/i/item/9789241548205

[9] Hobfoll SE , Watson PJ , Bell CC , Bryant RA , Brymer MJ , Friedman MJ , et al. Five essential elements of immediate and mid‐term mass trauma intervention: empirical evidence. Psychiatry. 2009;7:221–42. 10.1176/foc.7.2.foc221

[10] George S , Everly J , Lating JM . The Johns Hopkins guide to psychological first aid. 2nd ed. Baltimore: Johns Hopkins University Press; 2022.

[11] Dieltjens T , Moonens I , Van Praet K , De Buck E , Vandekerckhove P . A systematic literature search on psychological first aid: lack of evidence to develop guidelines. PLoS One. 2014;9:e114714. 10.1371/journal.pone.0114714 25503520 PMC4264843

[12] Minihan E , Gavin B , Kelly BD , McNicholas F . COVID‐19, mental health and psychological first aid. Ir J Psychol Med. 2020;37:259–63. 10.1017/ipm.2020.41 32404221 PMC7468678

[13] Becker LA , Oxman AD. Overviews of reviews. In: Higgins JP , Green S editors. Cochrane handbook for systematic reviews of interventions. Chichester: John Wiley & Sons, Ltd. 2008. p. 607–31. 10.1002/9780470712184.ch22

[14] Lu C , Lu T , Ge L , Yang N , Yan P , Yang K . Use of AMSTAR‐2 in the methodological assessment of systematic reviews: protocol for a methodological study. Ann Transl Med. 2020;8:652. 10.21037/atm-20-392a 32566589 PMC7290613

[15] Wang L , Norman I , Xiao T , Li Y , Leamy M . Psychological first aid training: a scoping review of its application, outcomes and implementation. Int J Environ Res Public Health. 2021;18:4594. 10.3390/ijerph18094594 33926108 PMC8123604

[16] Mascie Taylor N , Moji K . Pandemics. Journal for Peace and Nuclear Disarmament. 2021;4:47–59. 10.1080/25751654.2021.1880769

[17] Shea BJ , Reeves BC , Wells G , Thuku M , Hamel C , Moran J , et al. AMSTAR 2: a critical appraisal tool for systematic reviews that include randomised or non‐randomised studies of healthcare interventions, or both. BMJ. 2017;358:j4008. 10.1136/bmj.j4008 28935701 PMC5833365

[18] Page MJ , Moher D , Bossuyt PM , Boutron I , Hoffmann TC , Mulrow CD , et al. PRISMA 2020 explanation and elaboration: updated guidance and exemplars for reporting systematic reviews. BMJ. 2020;372:n160. 10.1136/bmj.n160 PMC8005925 33781993

[19] Pollock A , Campbell P , Cheyne J , Cowie J , Davis B , McCallum J , et al. Interventions to support the resilience and mental health of frontline health and social care professionals during and after a disease outbreak, epidemic or pandemic: a mixed methods systematic review. Cochrane Database Syst Rev. 2020;5:CD013779. 10.1002/14651858.CD013779 PMC8226433 33150970

[20] Yue JL , Yan W , Sun YK , Yuan K , Su SZ , Han Y , et al. Mental health services for infectious disease outbreaks including COVID‐19: a rapid systematic review. Psychol Med. 2020;50:2498–513. 10.1017/S0033291720003888 33148347 PMC7642960

[21] Cénat JM , Mukunzi JN , Noorishad PG , Rousseau C , Derivois D , Bukaka J . A systematic review of mental health programs among populations affected by the Ebola virus disease. J Psychosom Res. 2020;131:109966. 10.1016/j.jpsychores.2020.109966 32087433

[22] Belfroid E , van Steenbergen J , Timen A , Ellerbroek P , Huis A , Hulscher M . Preparedness and the importance of meeting the needs of healthcare workers: a qualitative study on Ebola. J Hosp Infect. 2018;98:212–8. 10.1016/j.jhin.2017.07.001 28690117 PMC7114583

[23] Brown‐Johnson C , Vilendrer S , Heffernan MB , Winter S , Khong T , Reidy J , et al. PPE portraits‐a way to humanize personal protective equipment. J Gen Intern Med. 2020;35:2240–2. 10.1007/s11606-020-05875-2 32410125 PMC7224350

[24] Cao J , Wei J , Zhu H , Duan Y , Geng W , Hong X , et al. A study of basic needs and psychological wellbeing of medical workers in the fever clinic of a tertiary general hospital in Beijing during the COVID‐19 outbreak. Psychother Psychosom. 2020;89:252–4. 10.1159/000507453 32224612 PMC7179543

[25] Carvalho E , Castro P , León E , Del Río A , Crespo F , Trigo L , et al. Multi‐professional simulation and risk perception of health care workers caring for Ebola‐infected patients. Nurs Crit Care. 2019;24:256–62. 10.1111/nicc.12396 30460729

[26] Chang K , Gotcher DF , Chan M . Does social capital matter when medical professionals encounter the SARS crisis in a hospital setting. Health Care Manage Rev. 2006;31:26–33. 10.1097/00004010-200601000-00005 16493270

[27] Chen Q , Liang M , Li Y , Guo J , Fei D , Wang L , et al. Mental health care for medical staff in China during the COVID‐19 outbreak. Lancet Psychiatry. 2020;7:e15–6. 10.1016/S2215-0366(20)30078-X 32085839 PMC7129426

[28] Cheung EYL . An outbreak of fear, rumours and stigma: psychosocial support for the Ebola virus disease outbreak in West Africa. Intervention. 2015;13:70–6.

[29] Cunningham T , Rosenthal D , Catallozzi M . Narrative medicine practices as a potential therapeutic tool used by expatriate Ebola caregivers. Intervention. 2017;15:106–19.

[30] De Jong J Strengthening evidence for the scaling of psychological first aid in humanitarian settings. Elrha. 2019 [cited 2022 Aug 12]. Available from: https://www.elrha.org/project/strengthening-evidence-scaling-psychological-first-aid-humanitarian-settings/

[31] Ferranti EP , Wands L , Yeager KA , Baker B , Higgins MK , Wold JL , et al. Implementation of an educational program for nursing students amidst the Ebola virus disease epidemic. Nurs Outlook. 2016;64:597–603. 10.1016/j.outlook.2016.04.002 27364913 PMC7111699

[32] Klomp RW , Jones L , Watanabe E , Thompson WW . CDC's multiple approaches to safeguard the health, safety, and resilience of Ebola responders. Prehosp Disaster Med. 2020;35:69–75. 10.1017/S1049023X19005144 31818341 PMC7113416

[33] Lee SH , Juang YY , Su YJ , Lee HL , Lin YH , Chao CC . Facing SARS: psychological impacts on SARS team nurses and psychiatric services in a Taiwan general hospital. Gen Hosp Psychistry. 2005;27:352–8. 10.1016/j.genhosppsych.2005.04.007 PMC7132375 16168796

[34] Son H , Lee WJ , Kim HS , Lee KS , You M . Examination of hospital workers' emotional responses to an infectious disease outbreak: lessons from the 2015 MERS Co‐V outbreak in South Korea. Disaster Med Public Health Prep. 2019;13:504–10. 10.1017/dmp.2018.95 30334501

[35] Schreiber M , Cates DS , Formanski S , King M . Maximizing the resilience of healthcare workers in multi‐hazard events: lessons from the 2014–2015 Ebola response in Africa. Mil Med. 2019;184:114–20. 10.1093/milmed/usy400 30901435

[36] Blake H , Bermingham F , Johnson G , Tabner A . Mitigating the psychological impact of COVID‐19 on healthcare workers: a digital learning package. Int J Environ Res Public Health. 2020;17:2997. 10.3390/ijerph17092997 32357424 PMC7246821

[37] Cole CL , Waterman S , Hunter ECM , Bell V , Greenberg N , Rubin GJ , et al. Effectiveness of small group cognitive behavioural therapy for anxiety and depression in Ebola treatment centre staff in Sierra Leone. Int Rev Psychiatry. 2021;33:189–97. 10.1080/09540261.2020.1750800 32301358

[38] Agyapong VIO . Coronavirus disease 2019 pandemic: health system and community response to a text message (Text4Hope) program supporting mental health in Alberta. Disaster Med Public Health Prep. 2020;14:e5–6. 10.1017/dmp.2020.114. PMC7198462 32317038

[39] Bäuerle A , Skoda EM , Dörrie N , Böttcher J , Teufel M . Psychological support in times of COVID‐19: the Essen community‐based CoPE concept. J Public Health (Bangkok). 2020;42:649–50. 10.1093/pubmed/fdaa053 PMC7188143 32307516

[40] Ho CS , Chee CY , Ho RC . Mental health strategies to combat the psychological impact of coronavirus disease 2019 (COVID‐19) beyond paranoia and panic. Ann Acad Med Singapore. 2020;49:155–60. 32200399

[41] Jung SJ , Jun JY . Mental health and psychological intervention amid COVID‐19 outbreak: perspectives from South Korea. Yonsei Med J. 2020;61:271–2. 10.3349/ymj.2020.61.4.271 32233168 PMC7105405

[42] Li W , Yang Y , Liu ZH , Zhao YJ , Zhang Q , Zhang L , et al. Progression of mental health services during the COVID‐19 outbreak in China. Int J Biol Sci. 2020;16:1732–8. 32226291 10.7150/ijbs.45120 PMC7098037

[43] Li J , Yang Z , Qiu H , Wang Y , Jian L , Ji J , et al. Anxiety and depression among general population in China at the peak of the COVID‐19 epidemic. World Psychiatry. 2020;19:249–50. 32394560 10.1002/wps.20758 PMC7214959

[44] Park SC , Park YC . Mental health care measures in response to the 2019 novel coronavirus outbreak in Korea. Psychiatry Investig. 2020;17:85–6. 10.30773/pi.2020.0058 PMC7047003 32093458

[45] Percudani M , Corradin M , Moreno M , Indelicato A , Vita A . Mental health services in Lombardy during COVID‐19 outbreak. Psychiatry Res. 2020;288:112980. 10.1016/j.psychres.2020.112980 32315881 PMC7152889

[46] Qiu JY , Zhou DS , Liu J , Yuan TF . Mental wellness system for COVID‐19. Brain Behav Immun. 2020;87:51–2. 10.1016/j.bbi.2020.04.032 32298801 PMC7153523

[47] Wang H , Li T , Gauthier S , Yu E , Tang Y , Barbarino P , et al. Coronavirus epidemic and geriatric mental healthcare in China: how a coordinated response by professional organizations helped older adults during an unprecedented crisis. Int Psychogeriatr. 2020;32:1117–20. 32268928 10.1017/S1041610220000551 PMC7184143

[48] Wang C , Pan R , Wan X , Tan Y , Xu L , Ho CS , et al. Immediate psychological responses and associated factors during the initial stage of the 2019 coronavirus disease (COVID‐19) epidemic among the general population in China. Int J Environ Res Public Health. 2020;17:1729. 32155789 10.3390/ijerph17051729 PMC7084952

[49] Wang Y , Zhao X , Feng Q , Liu L , Yao Y , Shi J . Psychological assistance during the coronavirus disease 2019 outbreak in China. J Health Psychol. 2020;25:733–7. 32301628 10.1177/1359105320919177

[50] Yao H , Chen JH , Zhao M , Qiu JY , Koenen KC , Stewart R , et al. Mitigating mental health consequences during the COVID‐19 outbreak: lessons from China. Psychiatry Clin Neurosci. 2020;74:407–8. 10.1111/pcn.13018 PMC7267425 32363746

[51] Zhou X . Psychological crisis interventions in Sichuan Province during the 2019 novel coronavirus outbreak. Psychiatry Res. 2020;286:112895. 10.1016/j.psychres.2020.112895 32120170 PMC7133610

[52] Yoon MK , Kim SY , Ko HS , Lee MS . System effectiveness of detection, brief intervention and refer to treatment for the people with post‐traumatic emotional distress by MERS: a case report of community‐based proactive intervention in South Korea. Int J Ment Health Syst. 2016;10:51. 10.1186/s13033-016-0083-5 27504141 PMC4976505

[53] Albott CS , Wozniak JR , McGlinch BP , Wall MH , Gold BS , Vinogradov S . Battle buddies: rapid deployment of a psychological resilience intervention for health care workers during the COVID‐19 pandemic. Anesth Analg. 2020;131:43–54. 10.1213/ANE.0000000000004912 32345861 PMC7199769

[54] Tze Ping NP , Shoesmith WD , James S , Nor Hadi NM , Boon Yau EK , Jiann Lin L . Ultra Brief Psychological Interventions for COVID‐19 Pandemic: Introduction of a Locally‐Adapted Brief Intervention for Mental Health and Psychosocial Support Service. Malays J Med Sci. 2020;27:51–6. 10.21315/mjms2020.27.2.6 PMC7409577 32788841

[55] Kazerooni AR , Amini M , Tabari P , Moosavi M Peer mentoring for medical students during COVID‐19 pandemic via a social media platform. Med Educ. 2020;54:762–3. 10.1111/medu.14206 PMC7267157 32353893

[56] Kohrt BA , Blasingame E , Compton MT , Dakana SF , Dossen B , Lang F , et al. Adapting the crisis intervention team (CIT) model of police–mental health collaboration in a low‐income, post‐conflict country: curriculum development in Liberia, West Africa. Am J Public Health. 2015;105:e73–80. 25602903 10.2105/AJPH.2014.302394 PMC4330847

[57] Ng SM , Chan THY , Chan CLW , Lee AM , Yau JKY , Chan CHY , et al. Group debriefing for people with chronic diseases during the SARS pandemic: strength‐focused and meaning‐oriented approach for resilience and transformation (SMART). Community Ment Health J. 2006;42:53–63. 16429250 10.1007/s10597-005-9002-y PMC7087702

[58] Garriga M , Agasi I , Fedida E , Pinzón‐Espinosa J , Vazquez M , Pacchiarotti I , et al. The role of mental health home hospitalization care during the COVID‐19 pandemic. Acta Psychiatr Scand. 2020;141:479–80. 10.1111/acps.13173 32279309 PMC7262322

[59] Poremski D , Subner SH , Lam GFK , Dev R , Mok YM , Chua HC , et al. Effective infection prevention and control strategies in a large, accredited, psychiatric facility in Singapore. Infect Control Hosp Epidemiol. 2020;41:1238–40. 32321623 10.1017/ice.2020.163 PMC7200837

[60] Shao Y , Shao Y , Fei JM . Psychiatry hospital management facing COVID‐19: From medical staff to patients. Brain Behav Immun. 2020;88:947. 10.1016/j.bbi.2020.04.018 32283291 PMC7146708

[61] Starace F , Ferrara M . COVID‐19 disease emergency operational instructions for Mental Health Departments issued by the Italian Society of Epidemiological Psychiatry. Epidemiol Psychiatr Sci. 2020;29:e116. 10.1017/S2045796020000372 32228737 PMC7163186

[62] Xiang YT , Zhao YJ , Liu ZH , Li XH , Zhao N , Cheung T , et al. The COVID‐19 outbreak and psychiatric hospitals in China: managing challenges through mental health service reform. Int J Biol Sci. 2020;16:1741–4. 10.7150/ijbs.45072 32226293 PMC7098035

[63] Liu S , Yang L , Zhang C , Xiang YT , Liu Z , Hu S , et al. Online mental health services in China during the COVID‐19 outbreak. Lancet Psychiatry. 2020;7:e17–8. 10.1016/S2215-0366(20)30077-8 32085841 PMC7129099

[64] Zhou X , Snoswell CL , Harding LE , Bambling M , Edirippulige S , Bai X , et al. The role of telehealth in reducing the mental health burden from COVID‐19. Telemed J E‐Health. 2020;26:377–9. 10.1089/tmj.2020.0068 32202977

[65] Decosimo CA , Hanson J , Quinn M , Badu P , Smith EG . Playing to live: outcome evaluation of a community‐based psychosocial expressive arts program for children during the Liberian Ebola epidemic. Glob Ment Health. 2019;6:e3. 10.1017/gmh.2019.1 PMC6521133 31143464

[66] Waterman S , Cole CL , Greenberg N , Rubin GJ , Beck A . A qualitative study assessing the feasibility of implementing a group cognitive‐behavioural therapy‐based intervention in Sierra Leone. BJPsych Int. 2019;16:31–4. 10.1192/bji.2018.7 31144684 PMC6520535

[67] Waterman S , Hunter ECM , Cole CL , Evans LJ , Greenberg N , Rubin GJ , et al. Training peers to treat Ebola centre workers with anxiety and depression in Sierra Leone. Int J Soc Psychiatry. 2018;64:156–65. 10.1177/0020764017752021 29432085

[68] Kamara S , Walder A , Duncan J , Kabbedijk A , Hughes P , Muana A . Mental health care during the Ebola virus disease outbreak in Sierra Leone. Bull World Health Organ. 2017;95:842–7. 10.2471/BLT.16.190470 29200525 PMC5710077

[69] Decosimo CA , Hanson JE , Boland CR , Slawson DL , Littleton MA , Quinn M . A process description of playing to live! A community psychosocial arts program during Ebola. J Soc Behav Health Sci. 2017;11:176–99.

[70] Weissbecker I , Roshania R , Cavallera V , Mallow M , Leichner A , Antigua J , et al. Integrating psychosocial support at Ebola treatment units in Sierra Leone and Liberia. Intervention. 2018;16:69.

[71] International Federation of Red Cross Red Crescent Societies . Lessons learned: psychosocial support in Ebola ‐ psychosocial support IFRC. 2016 [cited 2022 Aug 12]. Available from: https://pscentre.org/lessons-learned-psychosocial-support-ebola/

[72] United Nations Children's Fun d. Evaluation of UNICEF's response to the Ebola outbreak in West Africa 2014–2015. United Nations Children's Fund; 2016 [cited 2022 Aug 12]. Available from: https://reliefweb.int/report/liberia/evaluation-unicef-s-response-ebola-outbreak-west-africa-2014-2015-enesfr

[73] Mymin Kahn D , Bulanda JJ , Weissberger A , Jalloh S , Von Villa E , Williams A . Evaluation of a support group for Ebola hotline workers in Sierra Leone. Int J Cult. Ment Health. 2016;9:164–71.

[74] World Health Organization . Statement on the 1st meeting of the IHR Emergency Committee on the 2014 Ebola outbreak in West Africa. World Health Organization; 2014 [cited 2022 Aug 12. Available from: https://www.who.int/news/item/08-08-2014-statement-on-the-1st-meeting-of-the-ihr-emergency-committee-on-the-2014-ebola-outbreak-in-west-africa

[75] World Health Organization . MhGAP humanitarian intervention guide (MhGAP‐HIG): clinical management of mental, neurological and substance use conditions in humanitarian emergencies. Geneva: World Health Organization; 2015.

[76] Baron N . The “TOT”: a global approach for the training of trainers for psychosocial and mental health interventions in countries affected by war, violence and natural disasters. Intervention. 2006;4:108–25. 10.1097/01.WTF.0000237880.57276.9e

[77] Etard JF , Sow MS , Leroy S , Touré A , Taverne B , Keita AK , et al. Multidisciplinary assessment of post‐Ebola sequelae in Guinea (Postebogui): an observational cohort study. Lancet Infect Dis. 2017;17:545–52. 10.1016/S1473-3099(16)30516-3 28094208

[78] Denis‐Ramirez E , Sørensen KH , Skovdal M . In the midst of a ‘perfect storm’: unpacking the causes and consequences of Ebola‐related stigma for children orphaned by Ebola in Sierra Leone. Child Youth Serv Rev. 2017;73:445–53. 10.1016/j.childyouth.2016.11.025

[79] Yoshida K , Moriguchi S , Koda M , Oka T , Ueno F , Ikai‐Tani S , et al. Publication rates in English of abstracts presented at the annual meeting of the Japanese Society of Psychiatry and Neurology. Psychiatry Clin Neurosci. 2022;76:206–11. 10.1111/pcn.13351 35294087

[80] Collogan LK , Tuma F , Dolan‐Sewell R , Borja S , Fleischman AR . Ethical issues pertaining to research in the aftermath of disaster. J Trauma Stress. 2004;17:363–72. 10.1023/B:JOTS.0000048949.43570.6a 15633915

[81] O′Mathúna DP . Conducting research in the aftermath of disasters: ethical considerations. J Evid Based Med. 2010;3:65–75. 10.1111/j.1756-5391.2010.01076.x 21349047

